# PD-Impedance Combined Control Strategy for Capture Operations Using a 3-DOF Space Manipulator with a Compliant End-Effector

**DOI:** 10.3390/s20236739

**Published:** 2020-11-25

**Authors:** Guohua Kang, Qi Zhang, Jiaqi Wu, Han Zhang

**Affiliations:** College of Astronautics, Nanjing University of Aeronautics and Astronautics, Nanjing 210016, China; qizhangspx@163.com (Q.Z.); wjqjump@163.com (J.W.); hanzhangnuaa@foxmail.com (H.Z.)

**Keywords:** space manipulator, on-orbit servicing, contact dynamics, impedance control, compliant control

## Abstract

The contact force/torque between the end-effector of the space manipulator and the target spacecraft will reduce the efficiency and safety of the capture task. A capture strategy using PD-impedance combined control algorithm is proposed to achieve compliant contact between the chaser and target spacecraft. In order to absorb the impact energy, a spring-damper system is designed at the end-effector, and the corresponding dynamics model is established by Lagrange’s equation. Then a PD-impedance control algorithm based on steady-state force tracking error is proposed. Using this method, a compliant contact between the chaser and target spacecraft is realized while considering the dynamic coupling of the system. Finally, the general equation of the reference trajectory of the manipulator end-effector is derived according to the relative velocity and impact direction. The performance of the proposed capture strategy is studied by a co-simulation of MSC Adams and MATLAB Simulink in this paper. The results show that the contact plane at the end-effector of the manipulator can decelerate and detumble the target spacecraft. Besides, the contact force, relative velocity, and angular velocity all decrease to zero gradually, and the final stable state can be maintained for a prescribed time interval.

## 1. Introduction

With the development of space technology, space manipulators have played an important role in on-orbit service (OOS), such as on-orbit assembly, maintenance, docking, and space debris capture [1,2,3]. In general, the spacecraft capture mission can be divided into five phases: (a) orbital approach; (b) rendezvous; (c) robotic arm deployment; (d) pre-grasping; (e) grab operation [4]. This paper mainly studies the pre-grasping phase. The purpose of this phase is to reduce the distance, relative velocity, and angular velocity between the chaser and target spacecraft so that the end-effector of the manipulator can grab the target directly [5,6]. The impact force between the space manipulator and the target spacecraft will increase the risk and uncertainty of the capture mission. However, the accurate establishment of the manipulator dynamics model and the proper compliant control strategy can avoid the damage to the chaser and target spacecraft and improve the success probability [7,8].

In order to prevent damage to spacecraft caused by impact pulses, He J et al. [9] designed a hybrid serial-parallel manipulator with high stiffness and working space. They used the Newton-Euler method to establish the dynamics model but did not analyze the effect of the end-effector on the manipulator. Stolfi A et al. [10] designed a spring-damper end-effector and modeled dynamic equations using the Kane method. However, due to the difficulty of contact force measurement, the specific structure of the end-effector was ignored in the dynamic model. In fact, irregular end-effector structures may affect the contact forces directly, and non-rigid end-effector may even change the dynamics model. Hence, it is important to model the manipulator and the end-effector simultaneously [11]. Usually, the flexibility of the manipulator joint will cause the system vibration, which will change the dynamic model and affect the control accuracy of the end-effector. Therefore, Bottin M [12] and Doria A [13] et al. identified the joint stiffness using experimental modal analysis and proposed a vibration dynamic model of a serial manipulator. In order to simplify the analysis of the impact procedure, this paper assumes that the joints of the space manipulator are rigid and ignores their flexibility.

In the control field of space manipulators, impedance control is one of the main compliant control methods due to its good robustness to contact disturbance and environmental uncertainty [14,15,16]. In this approach, a virtual spring-damper system is established, and the impedance error is defined as the error between the balance position of the virtual spring and the control system. Then, the compliant contact is realized by the dynamic relationship between the position of the end-effector and the contact force [17,18]. One of the difficulties of the impedance control algorithm in practical application is the dynamic coupling between the spacecraft base and the manipulator [19,20]. Due to this characteristic, the planning and control of the space manipulator are much more complicated than those of the fixed-base manipulators [21]. Naohiro U [22] and Abbas K [23] et al. fixed the pose of the base platform in ground experiments. However, the motion of the manipulator in this way is different from that in space. Jia Q et al. [24] controlled the actuators of the base and the manipulator separately (the actuators of the base generally include momentum wheels and thrusters, etc., while the actuators of the space manipulator are joint motors). However, this approach is only suitable for systems where the motion of the manipulator has less impact on the base. In addition, the uncertainty of the end-effector contact force is also one of the factors affecting the control results [25]. Duan J et al. [26] assumed that the contact process was like the compression of a linear spring and identified the stiffness coefficient [27,28] by adaptive algorithms [29,30]. Xue C et al. [31] compensated the uncertainties in robotic manipulator dynamics through the radial basis functions neural networks (RBFNNs). Besides, using the disturbance observer is also one of the common methods. However, the actual contact process is influenced by the shape and material of the end-effector, and the microgravity collision test on the ground is also a great challenge [32]. It is difficult to model and identify the contact force accurately [33].

In this paper, an end-effector structure with an axis spring and a torsional spring is designed, and the dynamic of the whole manipulator system is modeled by Lagrange’s equation [34]. This end-effector can absorb the impact energy and gradually release the absorbed energy through impedance control of the manipulator joints. In terms of control law, the constraint equation of PD control parameters of the base is derived employing the dynamic model. Then the relationship between the steady-state tracking error of impedance force and the impact force is established. Finally, the joint impedance control law based on the reference trajectory is derived. The proposed compliant control strategy solves the problems of contact force estimation and dynamic coupling and is suitable for the actual space manipulator system.

This paper is organized as follows. Section 2 introduces the spring-damper system and derives the space manipulator dynamic model based on Lagrange’s equation. Section 3 describes the compliant capture control strategy, including PD control for the base and impedance control for the manipulator. Section 4 introduces the evaluation requirements of compliant control and shows the simulation results. Section 5 concludes the paper.

## 2. Space Manipulator Modeling

**Figure 1:**
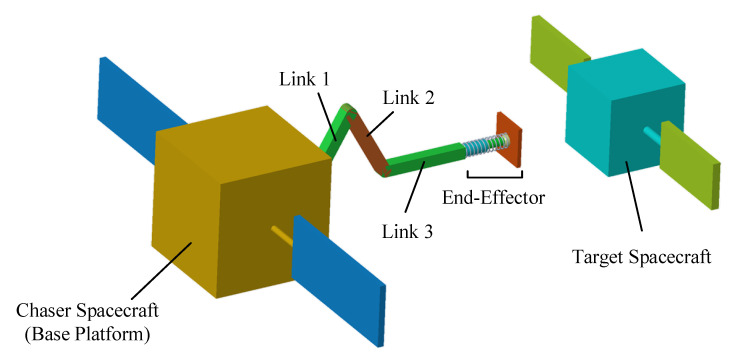
Multibody dynamic system of the chaser and target spacecraft.

The three degree of freedom (DOF) space manipulator studied in this paper consists of a base platform, three revolute joints, and a spring-damper end-effector, as shown in Figure 1. The manipulator is controlled by dynamic modeling to decelerate and detumble the target spacecraft as well as provide a good basis for grasping operation.

### 2.1. The End-Effector with a Spring-Damper System

**Figure 2:**
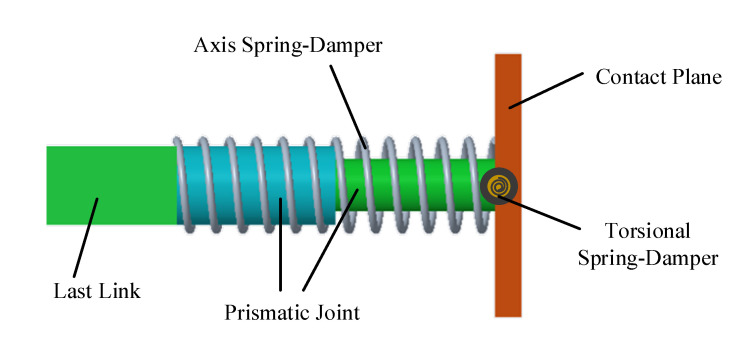
Schematic of the end-effector.

In order to absorb the impact energy and make the contact process behave like a spring-damper system, the end-effector structure is designed as shown in Figure 2. This end-effector is mainly composed of a prismatic joint, a contact plane, an axis spring, and a torsional spring, which is connected with the last joint of the manipulator [35]. Note that there is no active control unit in the end-effector. Hence, the impact energy is stored by the compression of the springs and is then released gradually through impedance control.

Assuming that the stiffness and damping coefficient of the above two springs are known, the impact force/torque can be calculated through offset and offset velocity of the springs. On this basis, the influence of contact force on the motion of base and joints can be obtained through the Jacobian matrix. Compared with the traditional methods, the complex environment modeling and disturbance compensation for the accurate estimation of the contact force are avoided. The contact force and torque can be calculated as follows:

(1) $$f_{e}=s_{as}\Delta x_{s}+s_{ad}\Delta {\dot{x}}_{s},$$

(2) $$T_{e}=s_{\mathrm{ts}}\Delta \theta_{s}+s_{\mathrm{td}}\Delta {\dot{\theta }}_{s},$$

where, $f_{e}$ and $T_{e}$ represent the impact force and torque, respectively; $s_{\mathrm{as}}$ and $s_{\mathrm{ad}}$ represent the stiffness and damping coefficient of the axis spring, respectively. $s_{\mathrm{ts}}$ and $s_{\mathrm{td}}$ represent the stiffness and damping coefficient of the torsional spring, respectively; ${\Delta x}_{s}$ and ${\Delta \theta }_{s}$ represent the offsets of axis spring and torsional spring, respectively; ${\Delta \dot{x}}_{s}$ and ${\Delta \dot{\theta }}_{s}$ represent the offset velocity of axis spring and torsional spring, respectively.

### 2.2. Manipulator and Contact Modeling

For a general space manipulator, the dynamic equation can be expressed as follows:

(3) $$\left[\begin{array}{cc} P_{b} & P_{bm}\\ P_{bm}^{T} & P_{n} \end{array}\right]\left[\begin{array}{c} {\ddot{X}}_{0}\\ \ddot{\theta } \end{array}\right]+\left[\begin{array}{c} c_{b}\\ c_{n} \end{array}\right]=\left[\begin{array}{c} F_{b}\\ \tau_{n} \end{array}\right]+\left[\begin{array}{c} J_{b}^{T}\\ J_{m}^{T} \end{array}\right]F_{e},$$

where, $P_{b}$ and $P_{bm}$ are coupling inertia matrices; $P_{n}$ denotes the inertia matrix of the manipulator; $X_{0}$ denotes the pose of the manipulator; $\theta =\left[\theta_{1},\theta_{2},\cdots ,\theta_{n}\right]$ denotes the joint angle; $c_{b}$ and $c_{m}$ are nonlinear terms of the base and manipulator, respectively; $F_{b}$ denotes the external force and torque on the base; $\tau_{n}$ denotes the joint torque; $J_{b}$ and $J_{m}$ represent the Jacobian matrices of the base and the manipulator, respectively; $F_{e}$ denotes the external force and torque applied on end-effector.

Suppose $\dot{q}={\left[{\dot{X}}_{0},\dot{\theta }\right]}^{T}$ and rewrite Equation (3) as follows:

(4) $$H(q)\ddot{q}+C(q,\dot{q})=F,$$

To further derive the nonlinear terms, Lagrange’s function is defined as follows:

(5) L=T+P,

where, T and P represent the kinetic energy and potential energy of the system, respectively. Since the space arm is in microgravity, it can be considered that P=0. Further, kinetic energy can be expressed as follows:

(6) $$L=\frac{1}{2}{\dot{q}}^{T}H\dot{q},$$

From Lagrange’s equation, we can get

(7) $$\frac{d}{dt}(\frac{\partial L}{\partial \dot{q}})-\frac{\partial L}{\partial q}=F,$$

Hence, the nonlinear terms can be expressed as follows

(8) $$C(q,\dot{q})=\dot{H}(q)\dot{q}-\frac{\partial }{\partial q}(\frac{1}{2}{\dot{q}}^{T}H\dot{q}),$$

For the space manipulator in Figure 1, only two-dimensional motion is considered in this paper. The dynamic parameters of each rigid body and the specific derivation results in Equations (4)–(8) are shown in Appendix A.

## 3. Design of Combined Impedance-PD Control Strategy

**Figure 3:**
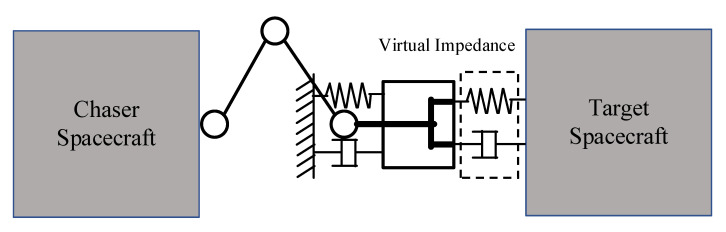
Impedance control model for the capture operation.

In the field of manipulator control, impedance control can interact well with the external environment, as shown in Figure 3. This method can make the manipulator end-effector behave like a spring-damper system to achieve compliant contact.

In space, the base platform is free-floating, and there is a characteristic of dynamic coupling between the base and the manipulator. Therefore, this problem further enhances the control requirements for the manipulator and increases the uncertainty of the control procedure. There are two main solutions at present: (a) Assume that the motion of the manipulator has very little effect on the base and therefore not control the base; (b) Control the base and the manipulator separately. The first method is suitable for systems with a large inertia ratio between the base and the manipulator. In the capture procedure, this method is usually not chosen because it is difficult to keep the base undisturbed. The second method is suitable for redundant manipulator systems, which can satisfy the pose requirements of the base and the end-effector, simultaneously. However, this method is easy to cause the oscillation of the manipulator system, and it is difficult to achieve high control accuracy. On the basis of the second method, the control strategy proposed in this paper employs impedance control for the manipulator joints while considering the effect of the base control on the joints.

**Figure 4:**
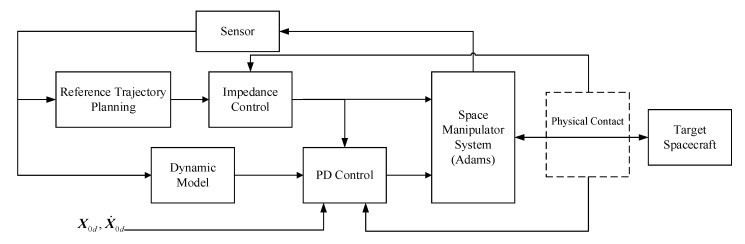
Schematic of the closed-loop PD-impedance combined control system.

This section will analyze the control procedure of the base and the space manipulator, respectively. PD control is adopted for the base and impedance control for the manipulator. The control block diagram is shown in Figure 4. Firstly, the manipulator dynamics model in Section 2.2 is employed to get the influence of $\tau_{n}$ and $F_{e}$ on the base pose. Then, the constraint equation of PD control parameters can be derived. Finally, the base pose requirements and the reference trajectory planning of the end-effector are achieved simultaneously.

### 3.1. The End-Effector with a Spring-Damper System

Usually, the spacecraft will carry many directional components (such as communication antenna, solar panel, etc.). Therefore, it is necessary to control the base pose not to change in a large range during the capture operations. Define the position and attitude of the base as $X_{0}={\left[x_{0},y_{0},\theta_{0}\right]}^{T}$, then the PD control law can be written as:

(9) $${\tilde{\ddot{X}}}_{0}=K_{p}(X_{0d}-X_{0})+K_{d}({\dot{X}}_{0d}-{\dot{X}}_{0}),$$

where, $K_{p}\in {\mathbb{R}}^{3\times 3}$ and $K_{d}\in {\mathbb{R}}^{3\times 3}$ are the control proportional and derivative gain matrices respectively; $X_{0d}$ and ${\dot{X}}_{0d}$ represent the desired position and velocity, respectively. In order to improve the control precision of the end-effector, $K_{p}$ and $K_{d}$ need to be constrained by the dynamic equation of the manipulator.

Expanding Equation (3) we can get

(10) $$\{\begin{array}{c} P_{b}{\ddot{X}}_{0}+P_{bm}\ddot{\theta }+c_{b}=F_{b}+J_{m}^{T}F_{e}\\ P_{bm}^{T}{\ddot{X}}_{0}+P_{n}\ddot{\theta }+c_{n}=\tau_{n}+J_{b}^{T}F_{e} \end{array},$$

Eliminating the angular acceleration $\ddot{\theta }$ of the joint:

(11) $$(P_{bm}^{T}-P_{n}P_{bm}^{-1}P_{b}){\ddot{X}}_{0}+P_{b}P_{bm}^{-1}(F_{b}+J_{m}^{T}F_{e}-c_{b})+c_{n}=\tau_{n}+J_{b}^{T}F_{e},$$

Suppose that the mass of the base is $M_{0}$ and the moment of inertia is $J_{0}$, the external generalized force and torque can be written as

(12) $$F_{b}=D_{0}{\tilde{\ddot{X}}}_{0},$$

(13) $$D_{0}=\left[\begin{array}{ccc} M_{0} & 0 & 0\\ 0 & M_{0} & 0\\ 0 & 0 & J_{0} \end{array}\right],$$

Substituting Equations (9) and (12) into Equation (11)

(14) $$K_{p}\Delta X_{0}+K_{d}\Delta {\dot{X}}_{0}={\left(P_{b}P_{bm}^{-1}D_{0}\right)}^{-1}(\tau_{n}+J_{b}^{T}F_{e}-c_{n}-(P_{bm}^{T}-P_{n}P_{bm}^{-1}P_{b}){\ddot{X}}_{0}-P_{b}P_{bm}^{-1}(J_{m}^{T}F_{e}-c_{b})),$$

To reduce the disturbance of the base motion to the manipulator, Equation (14) can be employed to get the gain matrices $K_{p}$ and $K_{d}$ according to joint torque and contact force.

### 3.2. Position-Based Impedance Control for Space Manipulator

**Figure 5:**
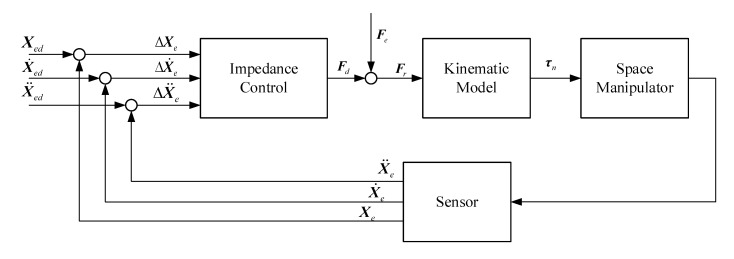
The position-based impedance control schematic.

Assuming that the desired pose of end-effector is $X_{ed}$ and the pose tracking error of end-effector is $\Delta X_{e}=X_{ed}-X_{e}$. The impedance control law of the manipulator can be expressed as follows:

(15) $$F_{d}=K_{c}\Delta X_{e}+B_{c}\Delta {\dot{X}}_{e}+M_{c}\Delta {\ddot{X}}_{e},$$

where, $F_{d}$ is the impedance force; $K_{c}$, $B_{c}$ and $M_{c}$ represent mass, damping, and stiffness matrices, respectively. Figure 5 shows the schematic diagram of impedance control after substituting the impedance control equation into Figure 4. Then, the following problem is how to plan the reference trajectory for the end-effector.

In Equations (1) and (2), each variable is independent [22]. Without loss of generality, take one dimension for example to derive the reference trajectory equation of the end-effector. From Figure 5 we can get

(16) $$f_{d}=f_{r}+f_{e},$$

The impact force $f_{e}$ can be expressed as

(17) $$f_{e}=g_{e}x_{e}+h_{e}{\dot{x}}_{e}+c_{e}=(g_{e}+h_{e}s)x_{e}+c_{e},$$

where s represents the Laplace transform. The derivation of Equation (17) is shown in Appendix B.

Substituting Equation (15) into Equation (17), yields

(18) $$f_{d}=f_{r}+(g_{e}+h_{e}s)(k(s)f_{d}+x_{ed})+c_{e},$$

(19) $$k(s)=\frac{1}{k_{e}+b_{e}s+m_{e}s^{2}},$$

After simplification, we can get

(20) $$(k_{e}+b_{e}s+m_{e}s^{2}-g_{e}-h_{e}s)f_{d}=(k_{e}+b_{e}s+m_{e}s^{2})(f_{r}+c_{e}+(g_{e}+h_{e}s)x_{ed}),$$

Then, the steady-state tracking error of the impedance force can be written as

(21) $$f_{dss}=\frac{k_{e}}{k_{e}-g_{e}}(f_{r}+c_{e}+g_{e}x_{ed}),$$

In Equation (21), the reference trajectory of the end-effector needs to satisfy the following equation to make steady-state error $f_{dss}$ converge to zero.

(22) $$x_{ed}=-\frac{f_{r}+c_{e}}{g_{e}},$$

Ignoring the spring offset $\Delta x_{s}$ and it can be seen that $c_{e}$ is proportional to the end-effector position $x_{e}$ according to Equation (A9). Reference [36] shows that the desired contact force $f_{r}$ is related to the collision velocity v and direction N. Assuming that the impact direction is the same as the relative velocity and substituting $f_{r}=G(v,\dot{v},N)$ into Equation (22), the reference trajectory can be written as follows.

(23) $$x_{ed}=\xi x_{e}+G(v_{ct}),$$

where, ξ represents the weight coefficient; $v_{ct}$ represents the relative velocity of the chaser and target spacecraft. In the pre-grasping phase, the contact force should be gradually reduced to zero and there is no manipulator joint torque when the system is stable. Therefore, the function G should satisfy the following requirements: (a) When the relative speed is large, a fast response function should be adopted; (b) When the relative speed is small, the end-effector pose should be kept as stable as possible to achieve compliant contact.

Then, the generalized Jacobian matrix can be employed to get joint torques through the planning of the reference trajectory.

(24) $$\tau_{n}=J_{g}^{T}F_{r},$$

where, $J_{g}$ is the generalized Jacobian matrix [37], which can be obtained by the kinematics equation. Furthermore, the desired trajectory of each joint can be obtained by integrating Equation (24). For redundant space manipulators, proper trajectory planning for joints can minimize base disturbance, kinetic energy, joint torques, and avoid singularities and physical joint limits [38,39]. This paper mainly studies the 3 DOF space manipulator, which has the minimum number of joints to meet the requirements of two-dimensional motion. The proposed algorithm is also applicable to the manipulators with higher degree of freedom.

## 4. Numerical Simulation

In this paper, MSC Adams software is employed to simulate the impact procedure between the chaser and target spacecraft. In this software, the dynamic model of the manipulator in space can be satisfied by creating outer-space conditions of micro-gravity. Besides, MATLAB/Simulink is used to realize PD-impedance combined control algorithm. The dynamic parameters of the space manipulator system are shown in Table 1. The stiffness coefficient $s_{as}$ and damping coefficient $s_{ad}$ of the axis spring are set as 500 N/m and 5 Ns/m, respectively. The stiffness coefficient $s_{ts}$ and damping coefficient $s_{td}$ of the torsional spring are 0.5 N/deg and 0.05 Ns/deg, respectively. In addition, the mass and inertia of the target spacecraft are also very important, and they are set as 115 kg and diag (11.54, 11.12, 6.11) kg∙m^2^, respectively.

### 4.1. Compliance Control Strategies Effectiveness Evaluation

The compliant contact control in the pre-grasping phase can be evaluated by the following five requirements:

(a) The distance between the end-effector and the target is null or within a prescribed tolerance (of the order of centimeters);
(b) The relative velocity and angular velocity of the target spacecraft are sufficiently small when the system is stable (respectively ≤0.5 cm/s and ≤0.05 deg/s);
(c) The translational displacements magnitude and angular displacement of the base are sufficiently small (respectively ≤5 cm and ≤1 deg);
(d) The contact force is sufficiently small (≤0.1 N);
(e) The stable state can maintain for a prescribed time interval (≥10 s).

### 4.2. Validity of Dynamic Equations

Note that the dynamics model in Section 2 is required for PD control and impedance control, and its accuracy is critical for compliant contact. In order to verify the correctness of the dynamic model, Equation (3) is used to calculate the acceleration and angular acceleration of the base and the angular acceleration of the joint. Then the calculated results are employed to compare with the simulation results in MSC Adams.

**Figure 6:**
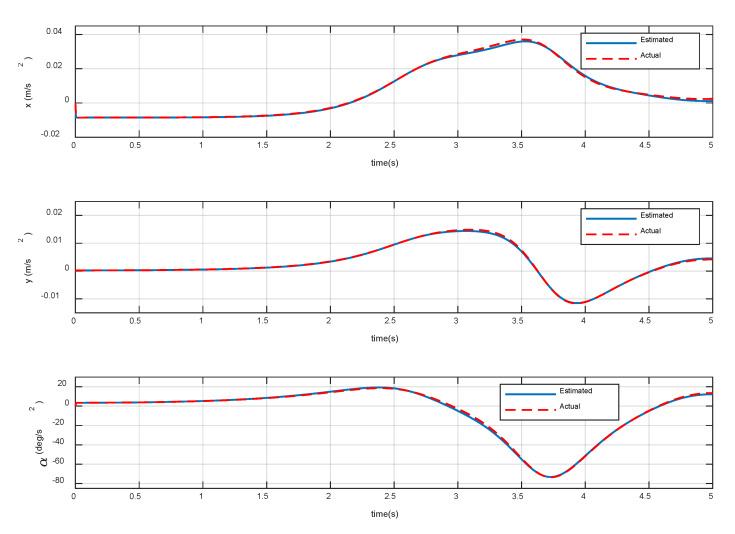
Variation of base acceleration and angular acceleration.

**Figure 7:**
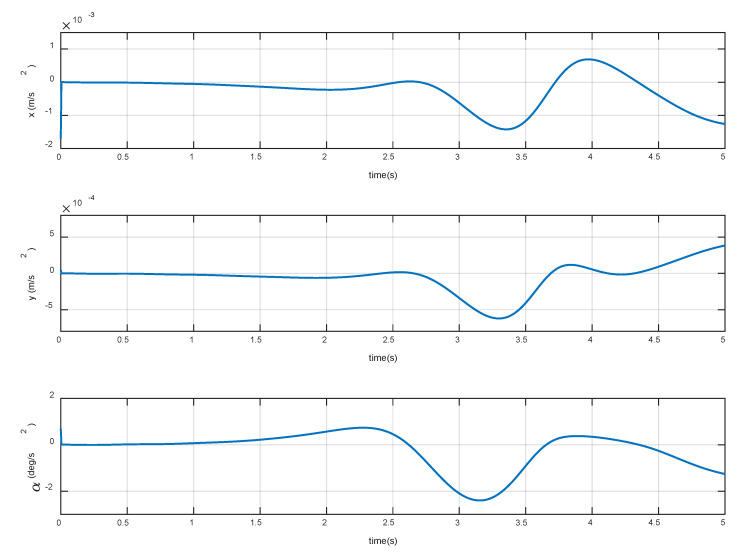
Errors of base acceleration and angular acceleration.

**Figure 8:**
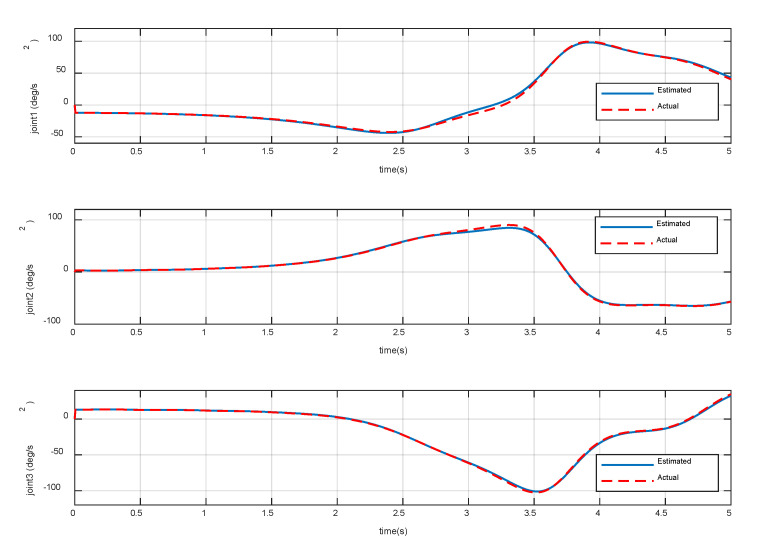
Variation of joint accelerations.

**Figure 9:**
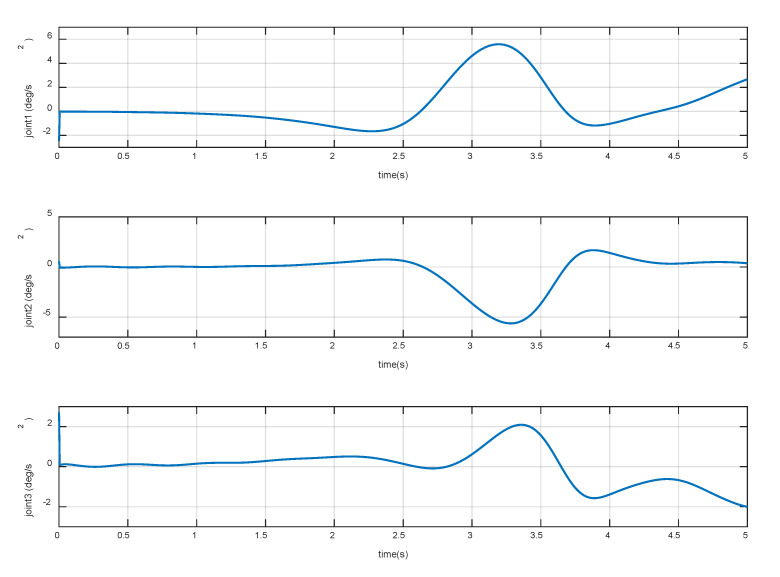
Errors of joint accelerations.

The constant torques (−0.3, 2.3, 1.2) Nm is continuously applied to the three joints of the manipulator, respectively. The variation of base accelerations and angular acceleration are shown in Figure 6, and the errors are shown in Figure 7. The joint angular accelerations are shown in Figure 8, and the errors are shown in Figure 9.

The simulation results show that the motion of the base and joint obtained by the dynamic model are very close to the true values. During the stationary phase, the errors are almost zero. During 3 s~5 s, the maximum acceleration errors of the base are 3.83% and 4.27%, the maximum error of angular acceleration is 3.28%, and the maximum errors of joint angular acceleration are 3.21%, 6.27%, and 6.10%, respectively. It can be seen that the overall errors are very small, and can be almost ignored in the relatively stable procedure. Therefore, it can be considered that the dynamic model established in Section 2 achieves high precision and satisfies the prescribed requirements of the control law.

### 4.3. Simulation Results of Contact Control

According to the description of the impact force function in Equation (23), this paper employs the exponential function to plan the reference trajectory as follows:

(25) $$G(v_{ct})=\{\begin{array}{ll} \lambda e^{\left\|v_{ct}\right\|}\frac{v_{ct}}{\left\|v_{ct}\right\|}, & \|v_{ct}\|\geq \varepsilon \\ 0, & \|v_{ct}\|<\varepsilon \end{array},$$

where, λ is the weight coefficient; ε is the relative velocity threshold.

To demonstrate the effectiveness of the proposed algorithm and the importance of the coupling characteristics of the base and joints, the performance of the independent control strategy and the proposed combined control strategy are simulated, respectively. The initial velocity and angular velocity of the target spacecraft are set as 0.17 m/s and 1.4 deg/s, respectively. In Equations (23) and (25), the weight coefficients are set as ξ=0.8 and λ=0.1, respectively; the relative speed threshold is set as ε=0.01. Besides, the mass, damping, and stiffness matrices for impedance control are set as $K_{c}=120\cdot diag(1,\;1,\;0.5)$, $B_{c}=100\cdot diag(1,\;1,\;0.5)$, and $M_{c}=1.5\cdot diag(1,\;1,\;0.5)$, respectively. The PD control parameters of independent control are $K_{p}=diag(5000,5000,\;1000)$ and $K_{d}=diag(1000,1000,\;500)$.

In the practical application of space manipulators, joint angles, velocities, and accelerations will be limited. Therefore, the corresponding constraint conditions are taken into account in the proposed PD-impedance combined algorithm. The constraint conditions of joints in simulation are as follows:

(26) $$\begin{array}{c} 90{}^{\circ}\leq \theta_{1}\leq 270{}^{\circ},-180{}^{\circ}\leq \theta_{2},\theta_{3}\leq 180{}^{\circ}\\ \left|{\dot{\theta }}_{i}\right|\leq 60{}^{\circ}/s,\left|{\ddot{\theta }}_{i}\right|\leq 80{}^{\circ}/s^{2},(i=1,2,3) \end{array}$$

**Figure 10:**
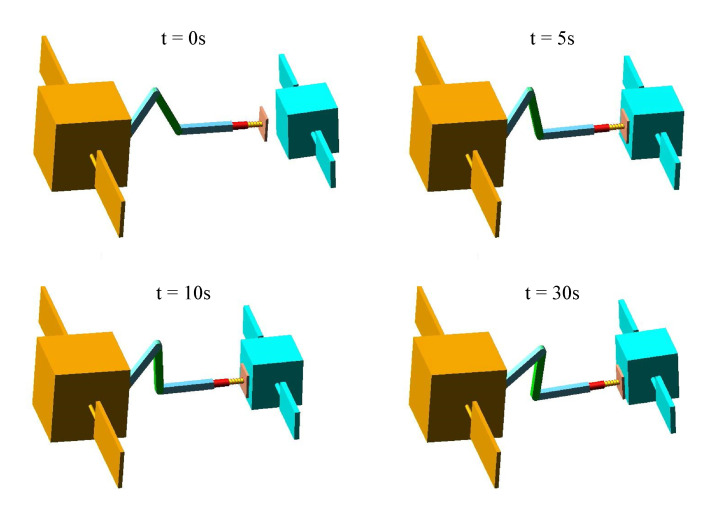
The chaser and target spacecraft during the contact phase using PD-impedance combined control strategy.

**Figure 11:**
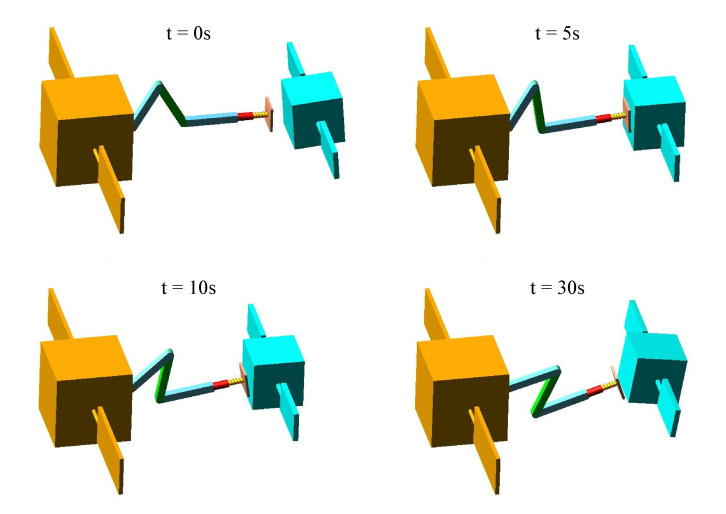
The chaser and target spacecraft during the contact phase using the PD control and impedance control independently.

Figure 10 and Figure 11 show the capture procedure of four different moments in Adams software using the above two control strategies. After the first contact, both methods can decelerate the target, but only the PD-impedance combined control can achieve the target detumbling. In addition, it can be seen that the impact energy is gradually released by the spring-damper system of the end-effector and the impedance control of the joint.

**Figure 12:**
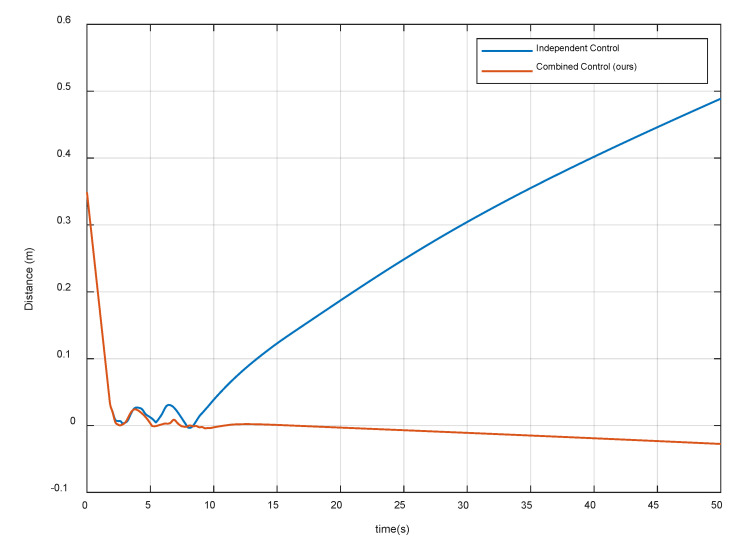
Distance magnitude between the contact plane and the target spacecraft surface.

**Figure 13:**
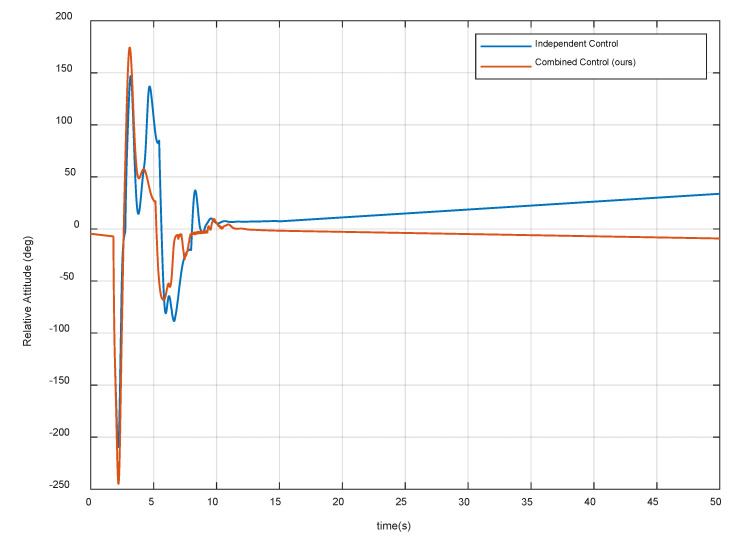
Relative attitude between the contact plane and the target spacecraft surface.

Figure 12 and Figure 13 show the distance magnitude and the relative attitude between the contact plane and the target spacecraft surface, respectively. The results show that the residual distance and attitude of the combined control strategy are significantly smaller than that of the independent control strategy. In the proposed combined control method, the system begins to oscillate after the first contact. According to Equation (25), when the relative velocity is less than the threshold ε, the G function can be changed to stabilize the system. Note that the contact plate at the end-effector is always attached to the surface of the target spacecraft during decelerating and detumbling. Thus, it satisfies the first compliance control requirements and provides a good relative pose for the grasping operation.

**Figure 14:**
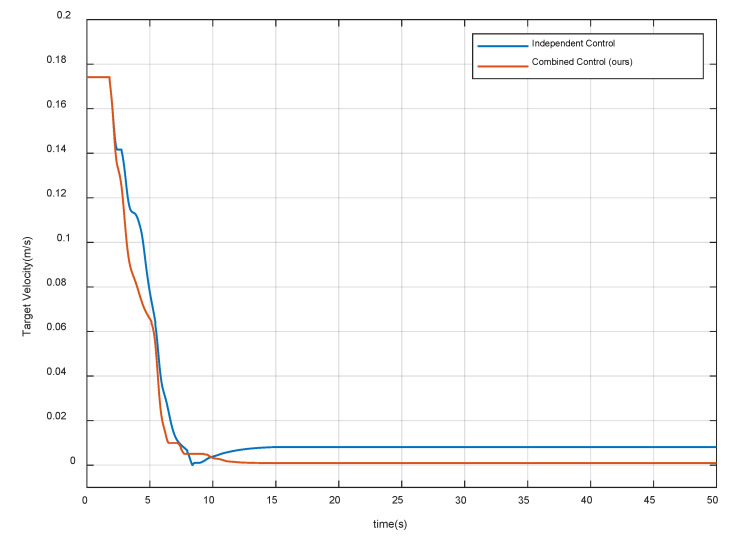
Velocity of the target spacecraft.

**Figure 15:**
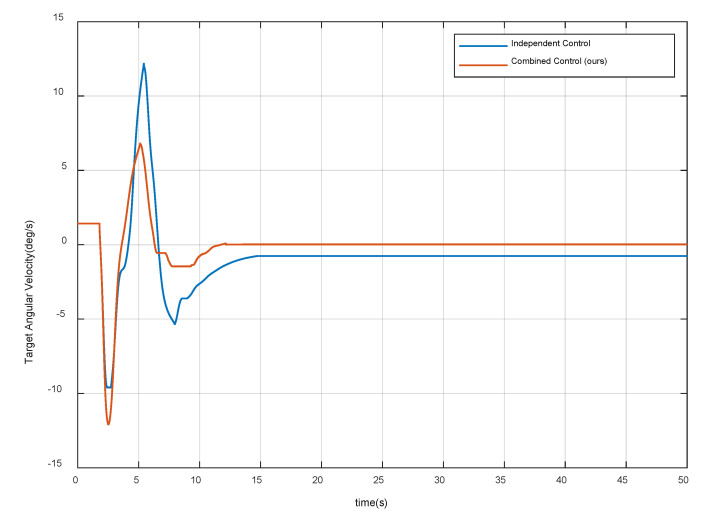
Angular velocity of the target spacecraft.

Figure 14 and Figure 15 show the velocity and angular velocity of the target spacecraft respectively. The results show that both strategies can effectively reduce the velocity of the target spacecraft, but the independent control strategy fails in target detumbling. Through the combined control strategy, when the system is stable, the velocity of the target spacecraft is even less than 0.1 cm/s and the angular velocity is less than 0.025 deg/s. Therefore, the second control requirement is met. Note that the compliant control procedure starts to stabilize and converge after 13 s, and stays in a stable state for more than 20 s. Thus, the proposed control algorithm also satisfies the fifth requirement.

**Figure 16:**
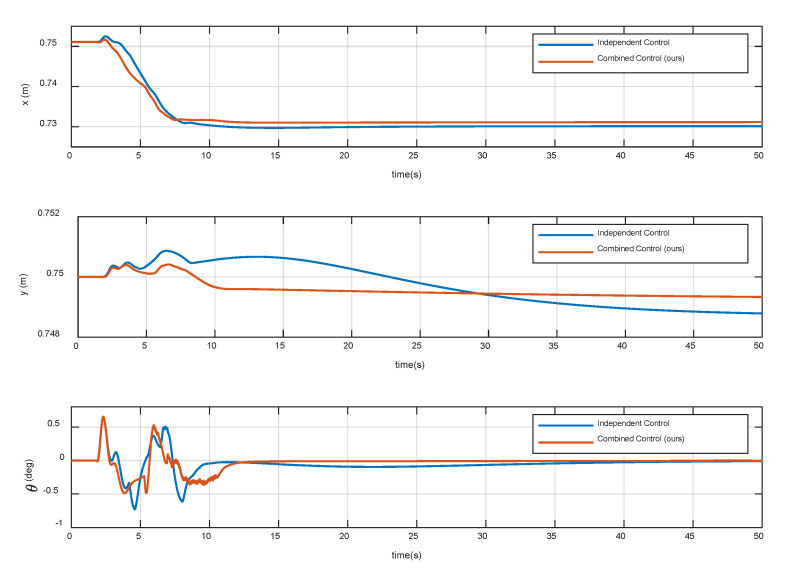
Variation of the base position and attitude.

**Figure 17:**
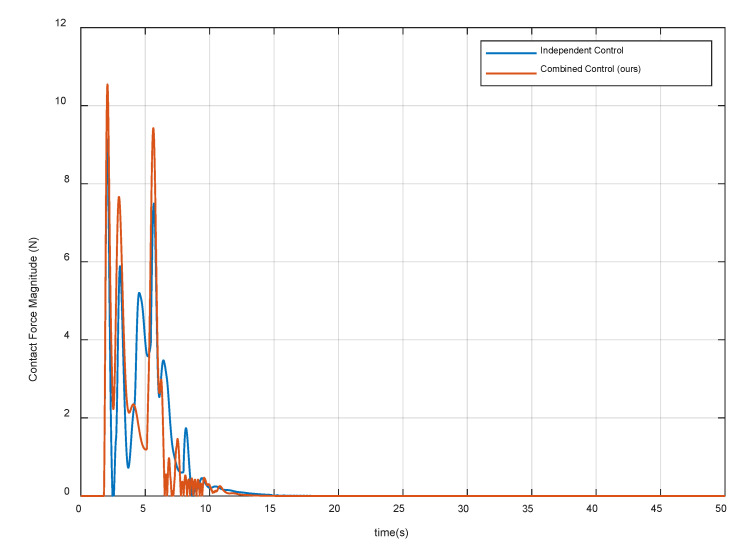
Variation of the impact force between the chaser and target spacecraft.

Figure 16 shows the position and attitude of the base. The results show that both methods can realize the position and attitude control of the base. During the compliant contact procedure, translational displacements magnitude of the base is less than 0.02 m, and the angular displacement is less than 0.75 deg, which meets the third control requirement. Figure 17 shows the variation of impact force. Note that the contact force starts to decrease gradually after reaching the maximum values. This indicates that the spring-damper system at the end-effector can store collision energy and the impedance control of the joint can release energy gradually. Besides, it can be seen that the contact forces and torque in the stable state are close to zero, thus the fourth control requirement is met. The above simulation results show that the independent control can satisfy some control requirements, but the combined control is better. Therefore, considering the disturbance of the base motion to the manipulator joints is necessary, which can improve the control accuracy and the success probability of the capture mission in the microgravity environment.

**Figure 18:**
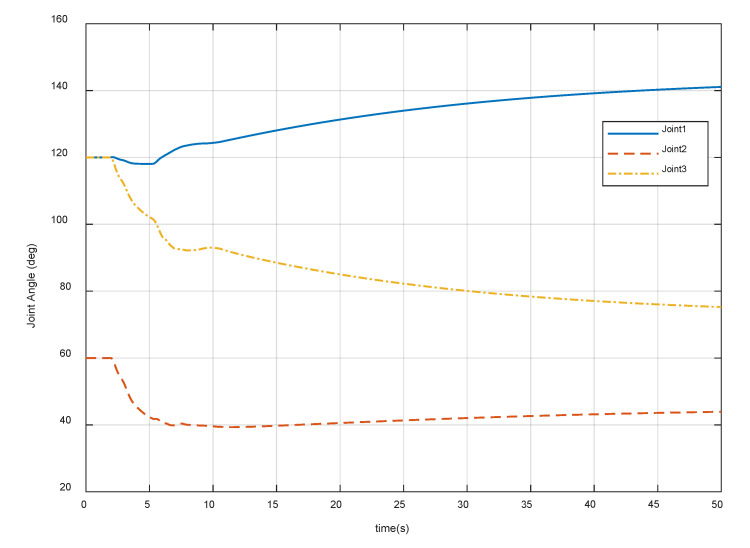
Variation of the manipulator joint angles.

**Figure 19:**
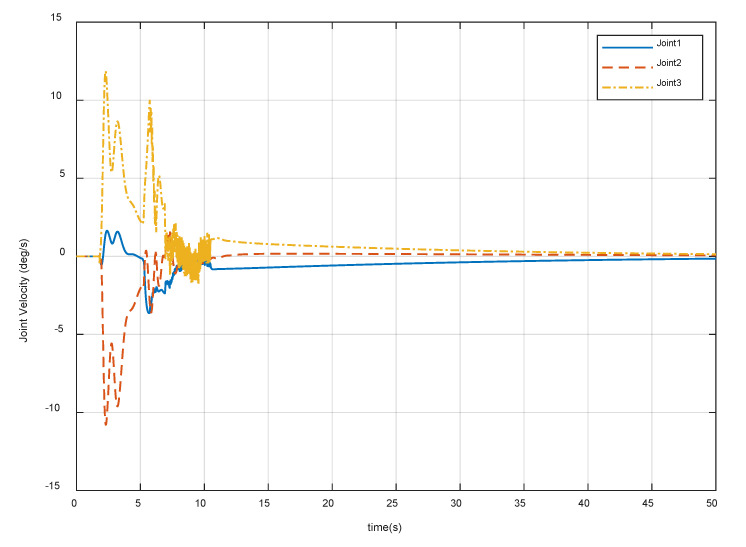
Variation of the manipulator joint velocities.

**Figure 20:**
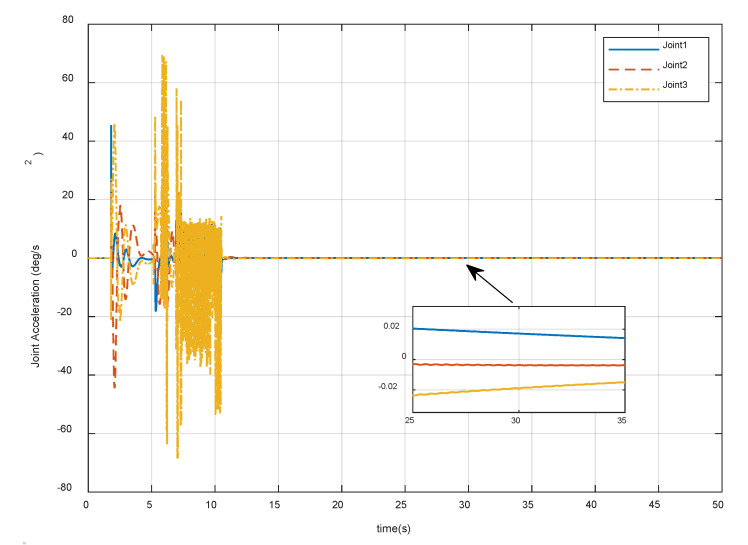
Variation of the manipulator joint accelerations.

After meeting all the requirements in Section 4.1, it is also important that the joint motion satisfies the actual physical constraints in Equation (26). Figure 18, Figure 19 and Figure 20 show the variation of the joint angles, velocities, and accelerations using the proposed combined control algorithm, respectively. It can be seen that the joint motion is sufficiently smooth and not out of bounds during the contact procedure. Note that there are also no joint singularities. The results display the compliance of the contact procedure and prove the effectiveness of the end-effector structure and the proposed control strategy.

## 5. Conclusions

In this paper, a 3-DOF space manipulator with a spring-damper system is designed and the corresponding dynamic model is established. On this basis, a compliant capture strategy based on PD-impedance combined control is proposed. This method solves the problem of low control precision caused by the dynamic coupling and the inaccurate estimation of the contact force. Firstly, the dynamic model is employed to derive the constraint equations of PD control parameters. Then the relationship between the steady-state force tracking error and the impact force is established, and the general equation of the reference end-effector trajectory is derived. Finally, MSC Adams and Simulink are employed to simulate the spring-damper end-effector and test the proposed compliant capture strategy. The results show that the spring-damper structure can realize the storage of impact energy and release energy gradually through joint motion. At the same time, the combined control strategy can decelerate and detumble the target spacecraft successfully. Besides, all the compliance control requirements are met, which proves the effectiveness of the proposed strategy. Besides, this method can be applied to different capture conditions by adjusting the control parameters and has good robustness.

## Figures and Tables

**Table 1:** Dynamic parameters of space manipulator.

Part	Mass (kg)	Length (m)	Moment of Inertia (kg·m^2^)
Base	364.07	0.9	diag (16.58, 15.57, 6.29)
Link 1	17.10	1	diag (4.33 × 10^−2^, 1.52, 1.52)
Link 2	17.10	1	diag (4.33 × 10^−2^, 1.52, 1.52)
Link 3	17.35	1.075	diag (4.47 × 10^−2^, 1.59, 1.59)
Prismatic Joint (Part 1)	0.88	0.3	diag (1.58 × 10^−3^, 5.42 × 10^−3^, 5.42 × 10^−3^)
Prismatic Joint (Part 2)	0.71	0.3	diag (5.83 × 10^−4^, 3.74 × 10^−3^, 3.74 × 10^−3^)
Contact Plane	3.75	0.05	diag (6.89 × 10^−2^, 0.1062, 6.89 × 10^−2^)

## Appendix A. Dynamic Model of the Space Manipulator

The space manipulator in Figure 1 consists of a total of 7 rigid bodies (including the base platform, three manipulator links, and the end-effector). Their poses are defined as $X_{i}={\left[r_{ix},r_{iy},\theta_{i}\right]}^{T}$, mass as $m_{i}$, and moment of inertia as $I_{ci}$, where i=0,1,⋯6. Since the prismatic joint has no rotating parts, their attitudes satisfy $\theta_{4}=\theta_{5}=0$. In addition, the distance from the center of mass of the base to the first link is defined as $b_{0}$, the length of the joint link is $l_{i}(i=1,2,3)$, and the length of the three rigid bodies at the end-effector (including two parts of the prismatic joint and the contact plane) is defined as $l_{i}(i=4,5,6)$.

Further, the kinetic energy of each rigid body can be expressed as

(A1) $$E_{ki}=\frac{1}{2}{\dot{X}}_{i}^{T}\left[\begin{array}{ccc} m_{i} & 0 & 0\\ 0 & m_{i} & 0\\ 0 & 0 & I_{ci} \end{array}\right]{\dot{X}}_{i}\;\;\;(i=0,1,2\cdots 6),$$

Therefore, Lagrange’s function in Equation (5) can be written as

(A2) $$L=\sum_{i=0}^{6}E_{ki},$$

Note that H is a symmetric matrix. Substituting Equation (A2) into Equations (7) and (8)

$H_{11}={\bar{m}}_{06}$
$H_{12}=0$
$H_{13}=-({\bar{m}}_{16}b_{0}s_{0}+(0.5m_{1}+{\bar{m}}_{26})l_{1}s_{01}+(0.5m_{2}+m_{36})l_{2}s_{02}+h_{p}s_{03}+0.5m_{6}l_{6}s_{06})$
$H_{14}=-((0.5m_{1}+{\bar{m}}_{26})l_{1}s_{01}+(0.5m_{2}+m_{36})l_{2}s_{02}+h_{p}s_{03}+0.5m_{6}l_{6}s_{06})$
$H_{15}=-((0.5m_{2}+m_{36})l_{2}s_{02}+h_{p}s_{03}+0.5m_{6}l_{6}s_{06})$
$H_{16}=-(l_{p}s_{03}+0.5m_{6}l_{6}s_{06})$
$H_{22}={\bar{m}}_{06}$
$H_{23}={\bar{m}}_{16}b_{0}c_{0}+(0.5m_{1}+{\bar{m}}_{26})l_{1}c_{01}+(0.5m_{2}+{\bar{m}}_{36})l_{2}c_{02}+h_{p}c_{03}+0.5m_{6}l_{6}c_{04}$
$H_{24}=(0.5m_{1}+{\bar{m}}_{26})l_{1}c_{01}+(0.5m_{2}+{\bar{m}}_{36})l_{2}c_{02}+h_{p}c_{03}+0.5m_{6}l_{6}c_{04}$
$H_{25}=(0.5m_{2}+{\bar{m}}_{36})l_{2}c_{02}+h_{p}c_{03}+0.5m_{6}l_{6}c_{04}$
$H_{26}=l_{p}c_{03}+0.5m_{6}l_{6}c_{04}$
$\begin{array}{ll} H_{33} & ={\bar{m}}_{16}b_{0}^{2}+(0.25m_{1}+{\bar{m}}_{26})l_{1}^{2}+(0.25m_{2}+{\bar{m}}_{36})l_{2}^{2}+0.25m_{3}l_{3}^{2}+m_{4}{\left(l_{3}+0.5l_{4}\right)}^{2}+m_{5}(h_{k}\\ & -0.5l_{5}{)}^{2}+m_{6}h_{k}^{2}+0.25m_{6}l_{6}^{2}+(m_{1}+2{\bar{m}}_{26})b_{0}l_{1}c_{1}+(m_{2}+2{\bar{m}}_{36})(b_{0}l_{2}c_{12}+l_{1}l_{2}c_{2})+2h_{p}l_{2}c_{3}\\ & +2h_{p}b_{0}c_{13}+2h_{p}l_{1}c_{23}+m_{6}l_{6}(h_{k}c_{4}+b_{0}c_{14}+l_{1}c_{24}+l_{2}c_{34})+{\bar{I}}_{c06} \end{array}$
$\begin{array}{ll} H_{34} & =(0.25m_{1}+{\bar{m}}_{26})l_{1}^{2}+(0.25m_{2}+{\bar{m}}_{36})l_{2}^{2}+0.25m_{3}l_{3}^{2}+m_{4}{\left(l_{3}+0.5l_{4}\right)}^{2}+m_{5}{\left(h_{k}-0.5l_{5}\right)}^{2}\\ & +m_{6}h_{k}^{2}+0.25m_{6}l_{6}^{2}+(0.5m_{1}+{\bar{m}}_{26})b_{0}l_{1}c_{1}+(0.5m_{2}+{\bar{m}}_{36})(b_{0}l_{2}c_{12}+l_{1}l_{2}c_{2})+2h_{p}l_{2}c_{3}+\\ & h_{p}b_{0}c_{13}+2h_{p}l_{1}c_{23}+m_{6}l_{6}(h_{k}c_{4}+b_{0}c_{14}+l_{1}c_{24}+l_{2}c_{34})+{\bar{I}}_{c16} \end{array}$
$\begin{array}{ll} H_{35} & =(0.25m_{2}+{\bar{m}}_{36})l_{2}^{2}+0.25m_{3}l_{3}^{2}+m_{4}{\left(l_{3}+0.5l_{4}\right)}^{2}+m_{5}{\left(h_{k}-0.5l_{5}\right)}^{2}+m_{6}h_{k}^{2}+0.25m_{6}l_{6}^{2}+\\ & (0.5m_{2}+{\bar{m}}_{36})(b_{0}l_{2}c_{12}+l_{1}l_{2}c_{2})+2h_{p}l_{2}c_{3}+h_{p}b_{0}c_{13}+h_{p}l_{1}c_{23}+m_{6}l_{6}(h_{k}c_{4}+0.5b_{0}c_{14}+\\ & 0.5l_{1}c_{24}+l_{2}c_{34})+{\bar{I}}_{c26} \end{array}$
$\begin{array}{ll} H_{36} & =0.25m_{3}l_{3}^{2}+m_{4}{\left(l_{3}+0.5l_{4}\right)}^{2}+m_{5}{\left(h_{k}-0.5l_{5}\right)}^{2}+m_{6}h_{k}^{2}+0.25m_{6}l_{6}^{2}+h_{p}l_{2}c_{3}+h_{p}b_{0}c_{13}+\\ & h_{p}l_{1}c_{23}+m_{6}h_{k}l_{6}c_{4}+0.25m_{6}l_{6}(b_{0}c_{14}+l_{1}c_{24}+l_{2}c_{34})+{\bar{I}}_{c36} \end{array}$
$\begin{array}{ll} H_{44} & =(0.25m_{1}+{\bar{m}}_{26})l_{1}^{2}+(0.25m_{2}+{\bar{m}}_{36})l_{2}^{2}+0.25m_{3}l_{3}^{2}+m_{4}{\left(l_{3}+0.5l_{4}\right)}^{2}+m_{5}{\left(h_{k}-0.5l_{5}\right)}^{2}\\ & +m_{6}h_{k}^{2}+0.5m_{6}l_{6}^{2}+(m_{2}+2m_{36})l_{1}l_{2}c_{2}+2h_{p}(l_{2}c_{3}+l_{1}c_{23})+m_{6}l_{6}(h_{k}c_{4}+l_{1}c_{24}+l_{2}c_{34})+{\bar{I}}_{c16} \end{array}$
$\begin{array}{ll} H_{45} & =(0.25m_{2}+{\bar{m}}_{36})l_{2}^{2}+0.25m_{3}l_{3}^{2}+m_{4}{\left(l_{3}+0.5l_{4}\right)}^{2}+m_{5}{\left(h_{k}-0.5l_{5}\right)}^{2}+m_{6}h_{k}^{2}+0.25m_{6}l_{6}^{2}+\\ & (m_{2}+2{\bar{m}}_{36})l_{1}l_{2}c_{2}+2h_{p}l_{2}c_{3}+h_{p}l_{1}c_{23}+m_{6}l_{6}(h_{k}c_{4}+0.5l_{1}c_{24}+l_{2}c_{34})+{\bar{I}}_{c26} \end{array}$
$\begin{array}{ll} H_{46} & =0.25m_{3}l_{3}^{2}+m_{4}{\left(l_{3}+0.5l_{4}\right)}^{2}+m_{5}{\left(h_{k}-0.5l_{5}\right)}^{2}+m_{6}h_{k}^{2}+0.25m_{6}l_{6}^{2}+h_{p}l_{2}c_{3}+h_{p}l_{1}c_{23}+\\ & m_{6}l_{6}(h_{k}c_{4}+0.5l_{1}c_{24}+0.5l_{2}c_{34})+{\bar{I}}_{c36} \end{array}$
$\begin{array}{ll} H_{55} & =(0.25m_{2}+{\bar{m}}_{36})l_{2}^{2}+0.25m_{3}l_{3}^{2}+m_{4}{\left(l_{3}+0.5l_{4}\right)}^{2}+m_{5}{\left(h_{k}-0.5l_{5}\right)}^{2}+m_{6}h_{k}^{2}+0.25m_{6}l_{6}^{2}\\ & +2h_{p}l_{2}c_{3}+m_{6}l_{6}(h_{k}c_{4}+l_{2}c_{34})+{\bar{I}}_{c26} \end{array}$
$\begin{array}{ll} H_{56} & =0.25m_{3}l_{3}^{2}+m_{4}{\left(l_{3}+0.5l_{4}\right)}^{2}+m_{5}{\left(h_{k}-0.5l_{5}\right)}^{2}+m_{6}h_{k}^{2}+0.25m_{6}l_{6}^{2}+h_{p}l_{2}c_{3}+m_{6}l_{6}(h_{k}c_{4}\\ & +0.5l_{2}c_{34})+{\bar{I}}_{c36} \end{array}$

(A3) $$H_{66}=0.25m_{3}l_{3}^{2}+m_{4}{\left(l_{3}+0.5l_{4}\right)}^{2}+m_{5}{\left(h_{k}-0.5l_{5}\right)}^{2}+m_{6}h_{k}^{2}+0.25m_{6}l_{6}^{2}+m_{6}h_{k}l_{6}c_{4}+{\bar{I}}_{c36}$$

$\begin{array}{ll} C_{1} & =-{\bar{m}}_{16}b_{0}{\ddot{\theta }}_{0}c_{0}-(0.5m_{1}+{\bar{m}}_{26})l_{1}{\ddot{\theta }}_{01}c_{01}-(0.5m_{2}+{\bar{m}}_{36})l_{2}{\ddot{\theta }}_{02}c_{02}-{\bar{m}}_{56}\Delta {\dot{x}}_{s}{\dot{\theta }}_{03}s_{03}-h_{p}{\ddot{\theta }}_{03}c_{03}\\ & -0.5m_{6}l_{6}({\ddot{\theta }}_{4}s_{04}+{\ddot{\theta }}_{04}c_{04}) \end{array}$
$\begin{array}{ll} C_{2} & =-{\bar{m}}_{16}b_{0}{\ddot{\theta }}_{0}s_{0}-(0.5m_{1}+{\bar{m}}_{26})l_{1}{\ddot{\theta }}_{01}s_{01}-(0.5m_{2}+{\bar{m}}_{36})l_{2}{\ddot{\theta }}_{02}s_{02}-{\bar{m}}_{56}\Delta {\dot{x}}_{s}{\dot{\theta }}_{03}c_{03}-h_{p}{\ddot{\theta }}_{03}s_{03}\\ & -0.5m_{6}l_{6}({\ddot{\theta }}_{4}c_{04}-{\ddot{\theta }}_{04}s_{04}) \end{array}$
$\begin{array}{ll} C_{3} & =(m_{5}(2h_{k}-l_{5})+2m_{6}h_{k})\Delta {\dot{x}}_{s}{\dot{\theta }}_{03}+0.25m_{6}l_{6}^{2}{\ddot{\theta }}_{4}-{\bar{m}}_{16}b_{0}({\dot{r}}_{0x}c_{0}+{\dot{r}}_{0y}s_{0}){\dot{\theta }}_{0}-(0.5m_{1}+\\ & {\bar{m}}_{26})l_{1}(({\dot{r}}_{0x}c_{01}+{\dot{r}}_{0y}s_{01}){\dot{\theta }}_{01}+b_{0}s_{1}(2{\dot{\theta }}_{0}{\dot{\theta }}_{1}+{\ddot{\theta }}_{1}^{2}))-(0.5m_{2}+{\bar{m}}_{36})l_{2}(({\dot{r}}_{0x}c_{02}+{\dot{r}}_{0y}s_{02}){\dot{\theta }}_{02}+\\ & b_{0}s_{12}{\dot{\theta }}_{12}({\dot{\theta }}_{0}+{\dot{\theta }}_{02})+l_{1}s_{2}{\dot{\theta }}_{2}(2{\dot{\theta }}_{01}+{\dot{\theta }}_{2}))+({\bar{m}}_{56}\Delta {\dot{x}}_{s}c_{3}-h_{p}s_{3}{\dot{\theta }}_{3})l_{2}({\dot{\theta }}_{02}+{\dot{\theta }}_{03})-{\bar{m}}_{56}\Delta {\dot{x}}_{s}({\dot{r}}_{0x}s_{03}\\ & -{\dot{r}}_{0y}c_{03})-h_{p}({\dot{r}}_{0x}c_{03}+{\dot{r}}_{0y}s_{03}){\dot{\theta }}_{03}+({\bar{m}}_{56}\Delta {\dot{x}}_{s}c_{13}-h_{p}s_{13}{\dot{\theta }}_{13})b_{0}({\dot{\theta }}_{0}+{\dot{\theta }}_{03})+({\bar{m}}_{56}\Delta {\dot{x}}_{s}c_{23}\\ & -h_{p}s_{23}{\dot{\theta }}_{23})l_{1}({\dot{\theta }}_{01}+{\dot{\theta }}_{03})+0.5m_{6}l_{6}(h_{k}(c_{4}{\ddot{\theta }}_{4}-s_{4}{\dot{\theta }}_{4}({\dot{\theta }}_{03}+{\dot{\theta }}_{04}))-({\dot{r}}_{0x}c_{04}+{\dot{r}}_{0y}s_{04}){\dot{\theta }}_{04}+\\ & b_{0}(c_{14}{\ddot{\theta }}_{4}-s_{14}{\dot{\theta }}_{14}({\dot{\theta }}_{0}+{\dot{\theta }}_{04}))+l_{1}(c_{24}{\ddot{\theta }}_{4}-s_{24}{\dot{\theta }}_{24}({\dot{\theta }}_{01}+{\dot{\theta }}_{04}))+l_{2}(c_{34}{\ddot{\theta }}_{4}-s_{34}{\dot{\theta }}_{34}({\dot{\theta }}_{02}+{\dot{\theta }}_{04})))+I_{c6}{\ddot{\theta }}_{4} \end{array}$
$\begin{array}{ll} C_{4} & =(m_{5}(2h_{k}-l_{5})+2m_{6}h_{k})\Delta {\dot{x}}_{s}{\dot{\theta }}_{03}+0.25m_{6}l_{6}^{2}{\ddot{\theta }}_{4}-(0.5m_{1}+{\bar{m}}_{26})(l_{1}({\dot{r}}_{0x}c_{01}+{\dot{r}}_{0y}s_{01}){\dot{\theta }}_{01}+\\ & {\dot{\theta }}_{0}s_{1}{\dot{\theta }}_{1})-(0.5m_{2}+{\bar{m}}_{36})l_{2}(({\dot{r}}_{0x}c_{02}+{\dot{r}}_{0y}s_{02}){\dot{\theta }}_{02}+b_{0}s_{12}{\dot{\theta }}_{12}{\dot{\theta }}_{0}+l_{1}s_{2}{\dot{\theta }}_{2}({\dot{\theta }}_{01}+{\dot{\theta }}_{02}))+({\bar{m}}_{56}\Delta {\dot{x}}_{s}c_{3}\\ & -h_{p}s_{3}{\dot{\theta }}_{3})l_{2}({\dot{\theta }}_{02}+{\dot{\theta }}_{03})-{\bar{m}}_{56}\Delta {\dot{x}}_{s}({\dot{r}}_{0x}s_{03}-{\dot{r}}_{0y}c_{03})-h_{p}({\dot{r}}_{0x}c_{03}+{\dot{r}}_{0y}s_{03}){\dot{\theta }}_{03}+({\bar{m}}_{56}\Delta {\dot{x}}_{s}c_{13}\\ & -h_{p}s_{13}{\dot{\theta }}_{13})b_{0}{\dot{\theta }}_{0}+({\bar{m}}_{56}\Delta {\dot{x}}_{s}c_{23}-h_{p}s_{23}{\dot{\theta }}_{23})l_{1}({\dot{\theta }}_{01}+{\dot{\theta }}_{03})+0.5m_{6}h_{k}l_{6}(c_{4}{\ddot{\theta }}_{4}-s_{4}{\dot{\theta }}_{4}({\dot{\theta }}_{03}+{\dot{\theta }}_{04}))+\\ & 0.5m_{6}l_{6}(l_{2}(c_{34}{\ddot{\theta }}_{4}-s_{34}{\dot{\theta }}_{34}({\dot{\theta }}_{02}+{\dot{\theta }}_{04}))+l_{1}(c_{24}{\ddot{\theta }}_{4}-s_{24}{\dot{\theta }}_{24}({\dot{\theta }}_{01}+{\dot{\theta }}_{04}))-({\dot{r}}_{0x}c_{04}+{\dot{r}}_{0y}s_{04}){\dot{\theta }}_{04}\\ & -b_{0}s_{14}{\dot{\theta }}_{14}{\dot{\theta }}_{0})+I_{c6}{\ddot{\theta }}_{4} \end{array}$
$\begin{array}{ll} C_{5} & =(m_{5}(2h_{k}-l_{5})+2m_{6}h_{k})\Delta {\dot{x}}_{s}{\dot{\theta }}_{03}+0.25m_{6}l_{6}^{2}{\ddot{\theta }}_{4}-(0.5m_{2}+{\bar{m}}_{36})l_{2}(({\dot{r}}_{0x}c_{02}+{\dot{r}}_{0y}s_{02}){\dot{\theta }}_{02}+\\ & b_{0}s_{12}{\dot{\theta }}_{12}{\dot{\theta }}_{0}+l_{1}s_{2}{\dot{\theta }}_{2}{\dot{\theta }}_{01})+({\bar{m}}_{56}\Delta {\dot{x}}_{s}c_{3}-h_{p}s_{3}{\dot{\theta }}_{3})l_{2}({\dot{\theta }}_{02}+{\dot{\theta }}_{03})-{\bar{m}}_{56}\Delta {\dot{x}}_{s}({\dot{r}}_{0x}s_{03}-{\dot{r}}_{0y}c_{03})\\ & -h_{p}({\dot{r}}_{0x}c_{03}+{\dot{r}}_{0y}s_{03}){\dot{\theta }}_{03}+({\bar{m}}_{56}\Delta {\dot{x}}_{s}c_{13}-h_{p}s_{13}{\dot{\theta }}_{13})b_{0}{\dot{\theta }}_{0}+({\bar{m}}_{56}\Delta {\dot{x}}_{s}c_{23}-h_{p}s_{23}{\dot{\theta }}_{23})l_{1}{\dot{\theta }}_{01}+\\ & 0.5m_{6}h_{k}l_{6}(c_{4}{\ddot{\theta }}_{4}-s_{4}{\dot{\theta }}_{4}({\dot{\theta }}_{03}+{\dot{\theta }}_{04}))-0.5m_{6}l_{6}({\dot{r}}_{0x}c_{04}+{\dot{r}}_{0y}s_{04}){\dot{\theta }}_{04}+0.5m_{6}l_{6}(l_{2}(c_{34}{\ddot{\theta }}_{4}\\ & -s_{34}{\dot{\theta }}_{34}({\dot{\theta }}_{02}+{\dot{\theta }}_{04}))-b_{0}s_{14}{\dot{\theta }}_{14}{\dot{\theta }}_{0}-l_{1}s_{24}{\dot{\theta }}_{24}{\dot{\theta }}_{01})+I_{c6}{\ddot{\theta }}_{4} \end{array}$

(A4) $$\begin{array}{ll} C_{6} & =(m_{5}(2h_{k}-l_{5})+2m_{6}h_{k})\Delta {\dot{x}}_{s}{\dot{\theta }}_{03}+0.25m_{6}l_{6}^{2}{\ddot{\theta }}_{4}+({\bar{m}}_{56}\Delta {\dot{x}}_{s}c_{3}-h_{p}s_{3}{\dot{\theta }}_{3})l_{2}{\dot{\theta }}_{02}-{\bar{m}}_{56}\Delta {\dot{x}}_{s}({\dot{r}}_{0x}s_{03}\\ & -{\dot{r}}_{0y}c_{03})-h_{p}({\dot{r}}_{0x}c_{03}+{\dot{r}}_{0y}s_{03}){\dot{\theta }}_{03}+({\bar{m}}_{56}\Delta {\dot{x}}_{s}c_{13}-h_{p}s_{13}{\dot{\theta }}_{13})b_{0}{\dot{\theta }}_{0}+({\bar{m}}_{56}\Delta {\dot{x}}_{s}c_{23}-h_{p}s_{23}{\dot{\theta }}_{23})l_{1}{\dot{\theta }}_{01}\\ & +0.5m_{6}l_{6}(h_{k}(c_{4}{\ddot{\theta }}_{4}-s_{4}{\dot{\theta }}_{4}({\dot{\theta }}_{03}+{\dot{\theta }}_{04}))-({\dot{r}}_{0x}c_{04}+{\dot{r}}_{0y}s_{04}){\dot{\theta }}_{04}-b_{0}s_{14}{\dot{\theta }}_{14}{\dot{\theta }}_{0}-l_{1}s_{24}{\dot{\theta }}_{24}{\dot{\theta }}_{01}\\ & -l_{2}s_{34}{\dot{\theta }}_{34}{\dot{\theta }}_{02})+I_{c6}{\ddot{\theta }}_{4} \end{array}$$

where

(A5) $$\theta_{ij}=\sum_{p=i}^{j}\theta_{p},{\bar{m}}_{ij}=\sum_{p=i}^{j}m_{p},{\bar{I}}_{cij}=\sum_{p=i}^{j}I_{cp}(i,j=0,1,\cdots 6),$$

(A6) $$s_{i}=\sin\theta_{i},,c_{i}=\cos\theta_{i},s_{ij}=\sin\theta_{ij},c_{ij}=\cos\theta_{ij}(i,j=0,1,\cdots 6),$$

(A7) $$h_{p}=1/2\cdot m_{3}l_{3}+1/2\cdot m_{4}(2l_{3}+l_{4})+1/2\cdot m_{5}(2h_{k}-l_{5})+m_{6}h_{k},$$

(A8) $$h_{k}=l_{3}+l_{5}+\Delta x_{s},$$

## Appendix B. The End-Effector Contact Force

In the fixed coordinate system of the manipulator, the position of the end-effector can be expressed as [40]

(A9) $$x_{e}=\frac{r_{4}m_{4}+(r_{4}+\Delta x_{s})m_{5}+(r_{4}+\Delta x_{s}+0.5(l_{5}+l_{6}))m_{6}}{{\bar{m}}_{46}},$$

The offset of the spring can be written as

(A10) $$\Delta x_{s}=\frac{{\bar{m}}_{46}(x_{e}-r_{4})-0.5m_{6}(l_{5}+l_{6})}{{\bar{m}}_{56}},$$

Taking the derivatives of Equation (A10) with respect to time

(A11) $$\Delta {\dot{x}}_{s}=\frac{{\bar{m}}_{46}}{{\bar{m}}_{56}}{\dot{x}}_{e},$$

Substituting Equations (A10) and (A11) into Equation (1)

(A12) $$\begin{array}{l} f_{e}=s_{as}\frac{{\bar{m}}_{46}(x_{e}-r_{4})-0.5m_{6}(l_{5}+l_{6})}{{\bar{m}}_{56}}+s_{ad}\frac{{\bar{m}}_{46}}{{\bar{m}}_{56}}{\dot{x}}_{e}\\ =(s_{as}+s_{ad}s)\frac{{\bar{m}}_{46}}{{\bar{m}}_{56}}x_{e}-\frac{{\bar{m}}_{46}}{{\bar{m}}_{56}}r_{4}-\frac{m_{6}}{2{\bar{m}}_{56}}(l_{5}+l_{6}) \end{array}$$

Hence, the parameters in Equation (17) can be written as

(A13) $$g_{e}=\frac{{\bar{m}}_{46}}{{\bar{m}}_{56}}s_{as},$$

(A14) $$h_{e}=\frac{{\bar{m}}_{46}}{{\bar{m}}_{56}}s_{ad},$$

(A15) $$c_{e}=-\frac{{\bar{m}}_{46}}{{\bar{m}}_{56}}r_{4}-\frac{m_{6}}{2{\bar{m}}_{56}}(l_{5}+l_{6})$$
